# New York Letter

**Published:** 1894-03

**Authors:** Wm. L. Russell

**Affiliations:** 101 West 93d Street.; New York


### CORRESPONDENCE.

### OUR NEW YORK LETTER.

NEW YORK, February 16, 1894.

SCURVY IN CHILDREN.

This was the subject of discussion at the meeting of the Academy, held last evening. A paper was read by Dr. W. P. Northrup, to whom credit is given of being the first to place the subject on its present footing. Observations on seventy-two cases were made by the different contributors to the meeting, and altogether it would seem that the disease, while comparatively new, occurs frequently enough, and under such improbable circumstances as to warrant a careful consideration of it by every practitioner.

Dr. Northrup said that the prevalence of scurvy among sailors and soldiers deprived of fresh water, vegetables and fruits had been recognized for many years. That it existed among the children of those in comfortable circumstances was not so well known. A German physician was the first to draw attention to this matter, by a case recorded in 1873. Three others were observed by a London physician, and recorded in the *Lancet* in 1878, and in 1882 a paper on the subject, based on observations of seven cases was read at the Congress at Copenhagen.

The first case seen by him was in 1889. It was not recognized until an autopsy had been made. In 1891 he saw another, a child surrounded by every comfort possible. The first child was a foundling, fifteen months old, which had been partly breast fed, and partly "slop" fed. When brought to the hospital its right leg and knee were swollen and tender. Later swelling and spunginess of the gums appeared, and hemorrhages into the skin and bowels occurred. At the autopsy pleuro-pneumonia was found, and extensive sub-periosteal hemorrhages in the femora and tibia. The other child had been fed for twelve months on a patent prepared babies' food, and had seemed to thrive. At the end of the period, however, the gums became swollen, spongy and tender, and the child ceased to move its right lower extremity, the thigh of which became enlarged and tender soon afterwards. A diagnosis of rheumatism was made, but no improvement occurring, he was called in consultation and, on the conditions of the gums and thigh, made a diagnosis of scurvy. The treatment consisted in removal to the country, and a change of diet to milk, beef-juice, potatoes and orange-juice. In five days the child was much improved, in ten the gums were better, and in thirty every vestige of disease had disappeared except slight swelling of the thigh.

The histories of eleven cases were narrated at the Congress in Washington, in 1891. The ages of eight of these were between eleven and eighteen months, the remaining three being two and a-half, two and eight-twelfths, and six years respectively. The large majority of the children had been fed exclusively on some kind of prepared food. One had been fed by a wet nurse who nursed her own child at the same time, and another had been fed for three months exclusively on beef-juice, which had been at first ordered by the physician because of diarrhea. The exact cause of the disease was not yet known. A bacillus had been observed, a culture of which injected into rabbits produced hemorrhages, but the matter was not yet decided. The symptom most uniformly observed was swollen, tender and bleeding gums. Bloody stools appeared in some cases, and swellings of the legs or thighs were quite common. The treatment which had been used in the cases recovering consisted mainly in a rational dietary with the addition of orange or lemon juice.

The diagnostic points were the condition of the gums, the subperiosteal hemorrhages causing swelling and tenderness, and usually anemia. The period between eleven and eighteen months was the usual age, and the large majority of cases were in children in comfortable circumstances who had been fed on a patent food or condensed milk. The disease frequently supervened upon rickets.

Dr. Henry Ling Taylor related the history of an interesting case which had come to him from the South, a diagnosis of either hip disease or spinal disease having been made. The child was eleven and a half months old, and had been fed exclusively on condensed milk for six or seven months. The first symptom noted had been an inability to sit up, a peculiar bent attitude being at first assumed, and afterwards the recumbent position. Then an apparent inability to move the legs or trunk appeared, and the right thigh became swollen and tender. The gums bled easily, and became swollen and purplish. Sleep was disturbed, the child was peevish and sensitive to touch or movement, and perspired freely. It had almost ceased to take food. The rectal temperature was 102.5°. An appearance of paralysis was noticed on examination, but the joints were found to move normally, and there was no change in the knee reflexes. The arms and head were moved freely, but the spine was rigid and bent, a projection at the first and second lumbar vertebræ being present. There were no symptoms of rickets, and the paralysis was not real. Scorbutus was suspected, and appropriate treatment adopted. Fresh milk and orange juice were given, and phosphate of soda as a laxative. Improvement was rapid. In two weeks the spine was flexible and straight, and a swelling of the ankle which had been present disappeared. The child soon began to sit up, to move its legs freely, and then went home. He had since read of its complete recovery. He had also learned that a neighbor's child, fed in the same way, had developed similar symptoms and died. The pseudo-paralysis in these cases was probably caused by the effusion into the muscles, and the painful traction on the affected periosteum.

Professor J. Lewis Smith said that the disease was very rare in his experience. He also referred to the fact that if there were no teeth, the gums might not be inflamed.

Professor Jacobi referred to prepared infant foods, as an "abomination," and added that no case of scurvy ever occurred when nutrition was normal. Since his attention had been drawn to the subject by Dr. Northrup's observations, he had seen several cases. Such cases had been formerly diagnosed as acute rickets and rheumatism, or as ulcerative stomatitis. These mistakes ought in future to be avoided.

Dr. L. Emmett Holt said that the records of the New York Infant Asylum, the Nursery and Child's Hospital, and the Babies'

Hospital showed that only one case of scurvy had appeared during the past five years, during which period all the milk used had been sterilized. The single case referred to had been fed for two months on malted milk exclusively. In a case seen by him in private practice, the parents were in good circumstances and had carefully cared for the child, traveling about with it from place to place for its health. It was well and robust-looking until eight months of age, when it became restless, and suffered pain when lifted. Ecchymosis appeared and the limbs were not moved well. The diet had been Mellin's Food. This was discontinued, milk being substituted, and the symptoms disappeared quickly. In the case seen in hospital, the diagnosis was sarcoma of the knee. The autopsy showed sub-periosteal hemorrhage. In other cases of which he had known, the diagnoses were, acute ostitis of the knee, for which a gypsum splint was applied; infantile paralysis, the physician claiming that he had discovered reaction of degeneration; rheumatism and rickets. All but one of his cases had come from the country, and most of these had been fed on Mellin's Food.

### PUERPERAL SEPSIS.

This subject, with special reference to its prophylaxis and treatment, was discussed at the last meeting of the County Society. Dr. R. A. Murray opened the discussion. He said that the procedure adopted by him consisted in ordering a preliminary bath for the patient, special attention being paid to the genitals. An enema and a vaginal injection (1-3000 bichloride or 2 per cent. creolin) were then given, and an antiseptic pad applied to the vulva, after which the patient was placed in a clean bed. Before examining, the hands were to be carefully cleaned and the finger lubricated with a creolin solution. The examinations should be few, but sufficient to follow the purposes of the labor. As soon as the child was born the antiseptic pad was to be reapplied, and left until the placenta was expressed. If it were necessary to introduce the hand into the uterus, or if hemorrhage occurred, an intra-uterine douche should be given. Otherwise no douche was necessary at any time, unless a necrotic odor was noticed, or symptoms of sepsis appeared. He stated that the mortality in the Maternity Hospital from 1875 to 1883, had been 4.17 per cent., and one year it was 6.67 per cent. From 1884 to 1888, the mortality had fallen to 1.6 per cent., that from sepsis being but .27 per cent. From 1890 to 1893, 957 labors recovered, and there were six deaths. Operative interference was necessary in one hundred and twenty-four cases, but not one case of sepsis occurred. This was surely the triumph of antiseptic midwifery. In his private practice he had had no case of sepsis for nineteen years. The cost for the pads and other things necessary to carry out the measures suggested was only about one dollar for each patient at the Maternity.

Dr. Grandin and Dr. Garrigues were of the opinion that when peritonitis occurred abdominal section might be resorted to, as it might save a life in what was otherwise an almost hopeless situation. Dr. Garrigues also referred to the fact that it was now just ten years ago since he had read his first paper on the subject of antiseptic midwifery, at which time many were inclined to take a humorous view of his suggestions.

### INTESTINAL PUTREFACTION.

Considerable original investigation in regard to this matter has been done by Dr. C. A. Hester of this city. In a paper read at a recent meeting of the Academy, he gave an account of his observations in cases of headache, urticaria, anemia, general malaise, and other conditions thought to be perhaps due to this cause. The presence of ethereal sulphates in the urine, the principal of which was indican, was examined for, and its relation to the clinical phenomena studied. The test for indican was so simple as to be easy of employment, and its presence in larger quantity than a mere trace was abnormal. Its relation to intestinal putrefaction was now pretty well recognized. In cases of occlusion from cancer it was found in large quantity, disappearing when the occlusion was relieved, and when a fistula was formed in the upper part of the small intestine it disappeared, to return again as soon as the fistula was closed. The only other condition in which it was found in any considerable quantity was that of suppuration without free drainage. It was derived from indol through the processes of putrefaction, and when aromatic substances, such as creosote or salol, were being used as therapeutic agents it would be increased. This was a point of importance, as these substances were used so frequently as intestinal antiseptics. It was difficult in practice to separate gastric and intestinal indigestion. The local symptoms of the latter might be slight and yet constitutional disturbance from the absorption of putrefactive products be pronounced. The local symptoms observed were, usually, flatulence, pain and excessive peristalsis. When such symptoms were present the changes in the urine accorded. In some cases, however, flatulence was not marked, or perhaps not noticeable, and yet indican was increased and constitutional symptoms occurred. Symptoms noticed by him were malaise, fatigue, twitchings of the muscles, cold and moist extremities, rapid and irregular heart, insomnia, hypochondriasis, loss of weight, frequent micturition, constituting a symptomatic group often classed as neurasthenia. The flatulence was sometimes removed by administering such food as was not liable to decompose. When no flatulence was present, broken down epithelium and the mucus of the bile might perhaps furnish the putrefactive products. This was probably the source of the large amount of indican found during starvation. The proper interpretation of the coincidence of the constitutional symptoms referred to, and the aromatic sulphates in the urine, was that the latter and toxic substances were probably formed coincidently during the process of putrefaction.

The treatment consisted in hygienic measures and the use of drugs. The intestinal antiseptics were irritants, and their even diffusion throught the gut was essential to effective action. Salol and resorcin could not be investigated, as they were themselves eliminated by the urine as ethereal sulphates. Salol was, however, effectual in removing the syptoms in some cases. Sodium salicylate in fifteen-grain doses three times daily was undoubtedly valuable; it acted as an irritant, however, in some persons, and less than ten grains was useless. The dietetic management consisted in the elimination of nearly all nitrogenous food except milk and meat.

Dr. L. C. Gray had found sub-gallate of bismuth a most useful remedy in these cases. Dr. Dana had been disappointed in the results obtained in cases of nervous disorder. His best results had been obtained with laxatives. Naphthaline had acted like magic in some cases of neurasthenia and migraine, but large doses were necessary. Dr. Wm. H. Thomson had used benzoate of soda in ten grain doses three times daily with much satisfaction. Bismuth and salol, he had found, usually prevented the diarrhea of typhoid fever.

Dr. L. Emmett Holt had seen many cases of children from one to three or four years old who were suffering from the effects of a diet composed too largely of carbohydrates, especially oatmeal. Such children quickly recovered on a diet of peptonized milk and raw or rare meat, with a calomel purge every three or four days.

WM. L. RUSSELL.

101 *West 93d Street.*

---

### THE EMERGENCY TREATMENT OF A TOOTHACHE.

Toothache is a little thing in the books, but many physicians would rather meet a burglar at the door on a dark night than a call to cure a bad toothache of several days' continuance ; a hypodermic of morphine only postpones the evil day, and usually the patient is respectfully referred to the dentist. The tooth should not be extracted while the jaw and gums are inflamed and the latter swollen, and it is the physician's duty to treat the case until the above conditions are removed. Always keep a small vial containing the following mixture : Chloroform, gtt. x. ; glycerine, gtt. x. ; sat. sol. ac. carbol., gtt. x. ; morphine, gr. j., with a small wad of absorbent cotton. If the offending tooth has a cavity or decayed surface, saturate a small pellet of cotton with the above mixture and put into the cavity or against the decayed surface as the case may be, never pack the cotton in, or the more is the trouble, but have the pellet small enough to enter without crowding. In most cases this will end the trouble. When the gums are swollen and tender paint two or three times, two minutes apart, with a four per cent. solution of cocaine. This time of year your patient may have been eating a good deal of fruit. The tongue and mucous membrane of the mouth are pale, sour stomach, and next day the toothache will return. Give ten grains of sub-carbonate of bismuth and ten grains of phenacetin at once and a similar dose before each of the three following meals, with a laxative if needed, and stop all fruit for a few days, and it will not return. The same powder every two hours, with cessation of fruit eating will stop the persistent, tormenting neuralgias so prevalent at this season.—*Jno. E. Weaver, M. D., of Rochester, N. Y., in Medical Record.*

